# Supplementary material for: Tutorial on Bayesian Functional Regression Using Stan
Source: Stat Med. 2025 Sep 14;44(20-22):e70265. doi: 10.1002/sim.70265 (PMC12433833; doi:10.1002/sim.70265)
Supplement: Supplementary file 1 — Data S1. Supporting Information. [file SIM-44-0-s001.zip › Supplementary materials/supp_code.pdf]

Supplementary code for Tutorial on Bayesian Functional Regression Using Stan

2025-07-25

Section 2: Scalar-on-function regression

Generate the data

```
n_num = 2000
T_num = 100
K_num = 6
basis_use = readRDS("basis_use.rds")


set.seed(1234)
eigenfunc=basis_use[2:(K_num+1),]
scores = matrix(rnorm(n_num*K_num,0,1),nrow = n_num, ncol = K_num)
mu_func = basis_use[1,]*1
W_mat =  matrix(mu_func,nrow = n_num,ncol = T_num,byrow = T) + scores %*% eigenfunc


true.beta.func=function(x){
  (-(-0.5+(x-0.5)^2)-0.416)*10
}

true.beta=true.beta.func((1:T_num)/T_num)
p_num = 1

data.X = rnorm(n_num,sd = 0.1)
true.X.effect = 0.1

linearPred.func = W_mat%*%matrix(true.beta,ncol=1)/length(true.beta)
linearPred.X = data.X%*%matrix(true.X.effect,ncol=1)
linearPred = linearPred.func+linearPred.X

logit.linearPred=exp(linearPred)/(1+exp(linearPred))

y.use = ifelse(runif(length(logit.linearPred))<logit.linearPred,1,0)
nt = ncol(W_mat)
tind = seq(0, 1, length.out = nt)
data.SoFR = data.frame(wmat = I(W_mat),
                       lmat = I(matrix(1/nt, ncol = nt, nrow = n_num)),
                       tmat = I(matrix(tind, ncol = nt, nrow = n_num, byrow = TRUE)))
```

Stan model

```
stan_model_SoFR = stan_model(model_code = "
data {
    int<lower=1> N_num;  // Total number of subjects
    int Y[N_num];  // Outcome variable
    int<lower=1> K_num;  // Number of scalar predictors
    // Design matrix for the scalar predictors
    matrix[K_num, N_num] Z_mat;
    int Kf; // Row number of the fixed effects design matrix
    int Kr; // Row number of the random effects design matrix
    matrix[Kf, N_num] X_mat_f; // Fixed effects design matrix
    matrix[Kr, N_num] X_mat_r; // Random effects design matrix
}

parameters {
    real<lower=0> sigma; // Smoothing parameter
    real eta_0; // Linear predictor intercept
    vector[Kf] betaf; // Fixed effects spline coefficients
    vector[Kr] betar; // Random effects spline coefficients
    vector[K_num] gamma; // Coefficients for scalar predictors
}


model {
    // Linear predictor
    vector[N_num] eta = rep_vector(0.0, N_num);
    eta += eta_0 + X_mat_f' * betaf + X_mat_r' * betar + Z_mat' * gamma;
    // Log-likelihood for the binary outcome
    target += bernoulli_logit_lpmf(Y | eta);
    // Set priors for the parameters
    target += normal_lpdf(betar | 0, sigma);
    target += inv_gamma_lpdf(sigma^2 | 0.001, 0.001);
}")
```

Stan data and sampling

```
smcon = mgcv:::smoothCon(s(tmat, by=lmat*wmat, bs="cc", k=10),data = data.SoFR,absorb.cons = TRUE,diago
randeff = mgcv::smooth2random(smcon[[1]], names(data.SoFR), type = 2)
X_mat_r = t(randeff$rand$Xr)
X_mat_f = t(randeff$Xf)

# Stan data list
data_stan = list()
data_stan[["N_num"]] = length(y.use)
data_stan[["Y"]] = c(y.use)
data_stan[["K_num"]] = NCOL(data.X)
data_stan[["Z_mat"]] = (t(data.X))
data_stan[["Kr"]]= NROW(X_mat_r)
data_stan[["Kf"]]= NROW(X_mat_f)
data_stan[["X_mat_r"]] = X_mat_r
```

```
data_stan[["X_mat_f"]] = X_mat_f


Bayes_fit_SoFR = sampling(stan_model_SoFR, data = data_stan, iter = 5000,warmup = 2000,chain=3,cores = [illegible]
```

Construct the estimated functional coefficient

```
object = s(tmat, by=lmat*wmat, bs="cc", k=10)
data = data.SoFR
knots = NULL
dk = ExtractData(object,data,knots)
splinecons = smooth.construct.cc.smooth.spec(object,dk$data,dk$knots)

Psi_mat = splinecons$X
S_mat = splinecons$S[[1]]
rank = splinecons$rank


#############################################################################
###     Calculate the X_mat, following the internal code of mgcv
#############################################################################

bymat=data.SoFR$lmat*data.SoFR$wmat
Psi_mat.new=matrix(1,nrow = prod(dim(bymat)), ncol=NCOL(Psi_mat))
for(i in 1:NROW(Psi_mat)){
  for(inx in ((i-1)*dim(bymat)[1]+1):(i*dim(bymat)[1])){
    Psi_mat.new[inx,]=Psi_mat[i,]
  }
}
Psi_mat = Psi_mat.new
maXX = norm(Psi_mat,type="I")^2
maS = norm(S_mat)/maXX
S_mat = S_mat / maS
eigendecomp = eigen(S_mat, symmetric = TRUE)
U_mat = eigendecomp$vectors
V_vec = eigendecomp$value[1:rank]

smlX <- as.numeric(bymat)*Psi_mat
ind <- 1:n_num
X_mat <- smlX[ind,,drop=FALSE]
for (i in 2:dim(data$wmat)[2]) {
  ind <- ind + n_num
  X_mat <- X_mat +smlX[ind,,drop=FALSE]
}
X_mat = X_mat %*% U_mat

#############################################################################
###     Reconstruct the spline coefficients 'beta.sample.untilde'
#############################################################################

V = rep(1, ncol(X_mat))
```

```r
V[1:rank] = sqrt(V_vec)
col.norm = colSums((X_mat)^2)
col.norm = col.norm / V^2
av.norm = mean(col.norm[1:rank])
for (i in (rank + 1):ncol(X_mat)) {
  V[i] = sqrt(col.norm[i] / av.norm)
}

post_sample = rstan::extract(Bayes_fit_SoFR)
beta.sample = t(cbind(post_sample$betar,post_sample$betaf) %*% randeff$trans.U)
beta.sample.untilde = (U_mat %*% diag(1 / V)) %*% beta.sample


##############################################################################
###     Reconstruct the estimated functional coefficient
##############################################################################

beta.post = apply(beta.sample.untilde, 2, function(x){x%*%t(splinecons$X)})

mean.curve.est=apply(beta.post,1,mean)
upper.curve.est=apply(beta.post,1,function(x){quantile(x,probs = 0.975)})
lower.curve.est=apply(beta.post,1,function(x){quantile(x,probs = 0.025)})

plotdata=data.frame(value=c(mean.curve.est,
                            upper.curve.est,
                            lower.curve.est),
                    xmat=c(rep(1: dim(data.SoFR$wmat)[2],3)),
                    Method=c(rep("Bayesian",dim(data.SoFR$wmat)[2]),
                           rep("Bayesian",dim(data.SoFR$wmat)[2]),
                           rep("Bayesian",dim(data.SoFR$wmat)[2])),
                    type=c(rep("Estimate",dim(data.SoFR$wmat)[2]),
                           rep("CI_upper",dim(data.SoFR$wmat)[2]),
                           rep("CI_lower",dim(data.SoFR$wmat)[2])))

library(ggplot2)
ggplot(plotdata,aes(y=value,x=xmat))+geom_line(aes(linetype=type,color=Method))+
  ylab("Functional effect")+xlab("Time (hour)")+
  #scale_x_continuous(breaks=seq(0,1,by=3))+
  scale_linetype_manual(values=c("twodash", "longdash","solid"),name="Line Type")+
  theme_minimal()
```

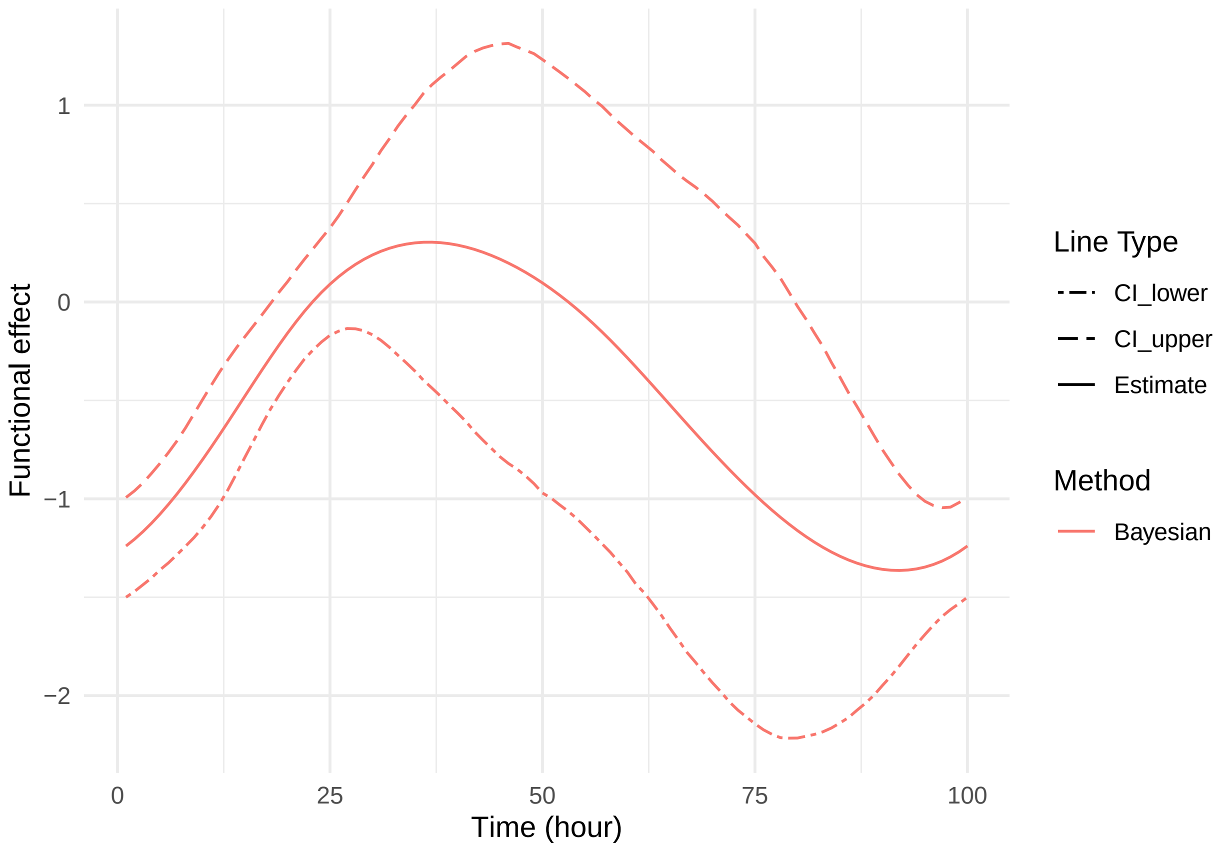

Compare with frequentist mgcv result

```
data.SoFR$y=c(y.use)
data.SoFR$X1=data.X
fit_freq1 = gam(y ~ s(tmat, by=lmat*wmat, bs="cc", k=10)+X1, data=data.SoFR, family="binomial")
fit_freq2 = gam(y ~ s(tmat, by=lmat*wmat, bs="cc", k=10)+X1, data=data.SoFR, family="binomial", method
plotfot=plot(fit_freq1)
```

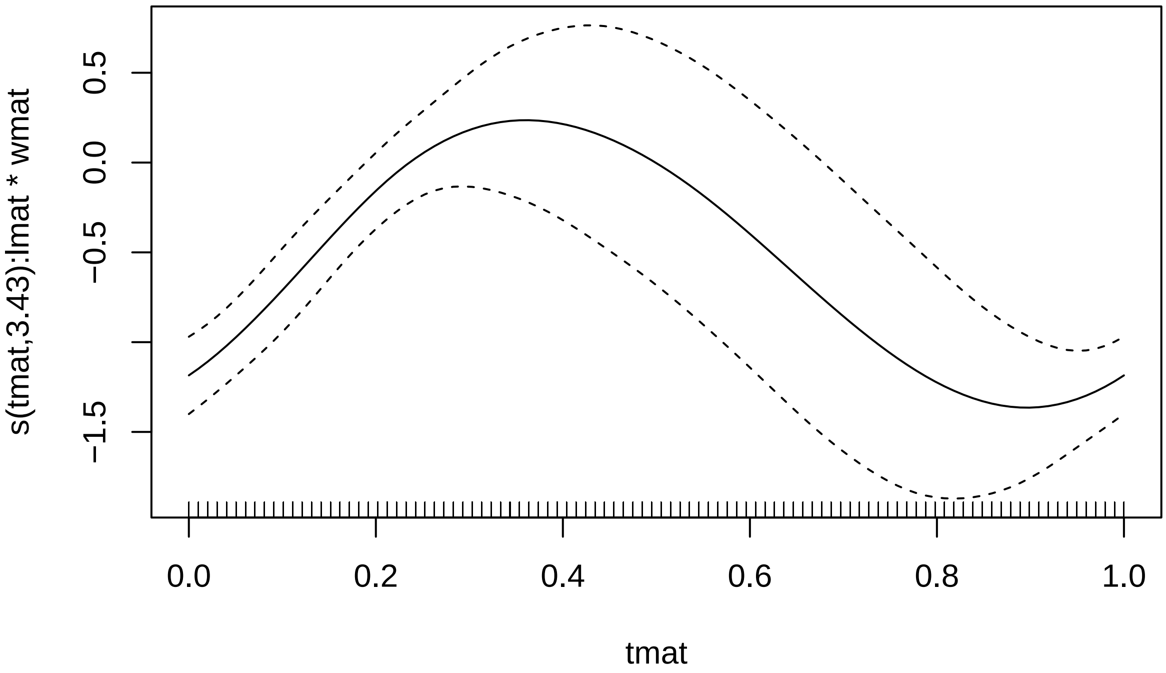


```
plotfot2=plot(fit_freq2,unconditional = TRUE)
```

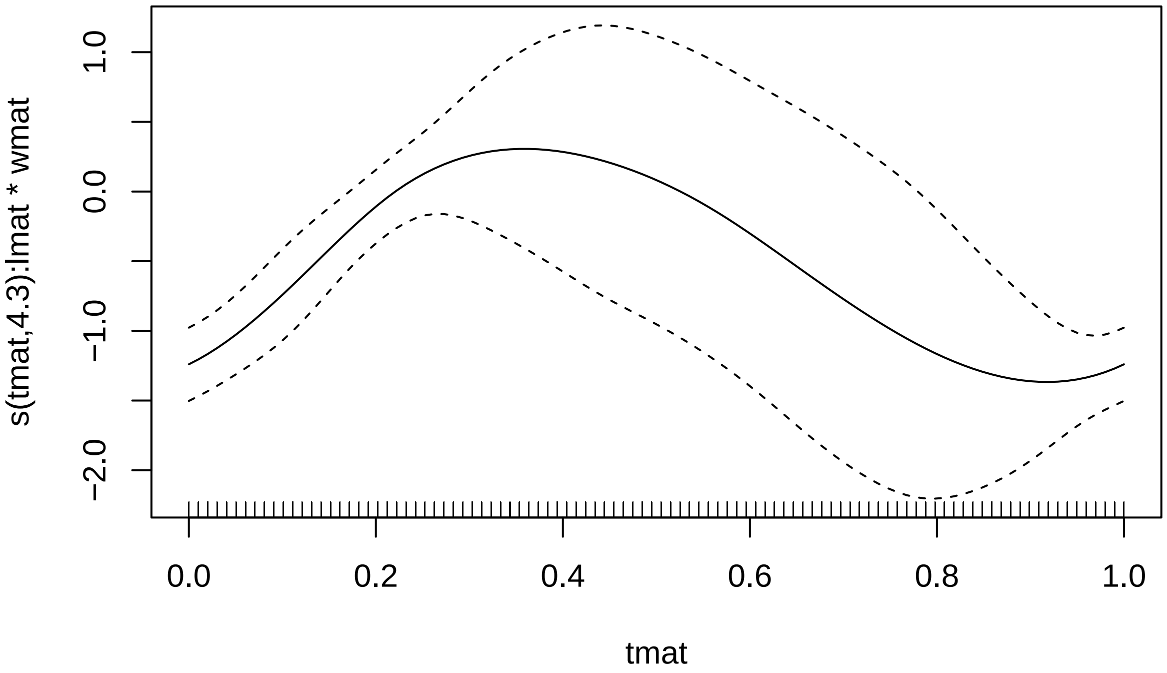


```
plotdata=data.frame(value=c(mean.curve.est,
                            upper.curve.est,
                            lower.curve.est,
                            plotfot[[1]]$fit,
                            plotfot[[1]]$fit+plotfot[[1]]$se,
                            plotfot[[1]]$fit-plotfot[[1]]$se,
                            plotfot2[[1]]$fit,
                            plotfot2[[1]]$fit+plotfot2[[1]]$se,
                            plotfot2[[1]]$fit-plotfot2[[1]]$se,
                            true.beta),
                    xmat=c(rep(1: T_num,3),
                           rep(plotfot[[1]]$x*100,7)),
                    Method=c(rep("Bayesian",T_num),
                           rep("Bayesian",T_num),
                           rep("Bayesian",T_num),
                           rep("Frequentist - GCV",100),
                           rep("Frequentist - GCV",100),
                           rep("Frequentist - GCV",100),
                           rep("Frequentist - REML",100),
                           rep("Frequentist - REML",100),
                           rep("Frequentist - REML",100),
                           rep("True",100)),
                    type=c(rep("Estimate",T_num),
                           rep("CI_upper",T_num),
                           rep("CI_lower",T_num),
                           rep("Estimate",100),
```

```r
                              rep("CI_upper",100),
                              rep("CI_lower",100),
                              rep("Estimate",100),
                              rep("CI_upper",100),
                              rep("CI_lower",100),
                              rep("Estimate",100)))

library(ggplot2)
ggplot(plotdata,aes(y=value,x=xmat))+geom_line(aes(linetype=type,color=Method))+
  ylab("Functional effect")+xlab("Time index")+
  #scale_x_continuous(breaks=seq(0,1,by=3))+
  scale_linetype_manual(values=c("twodash", "longdash","solid"),name="Line Type")+
  theme_minimal()
```

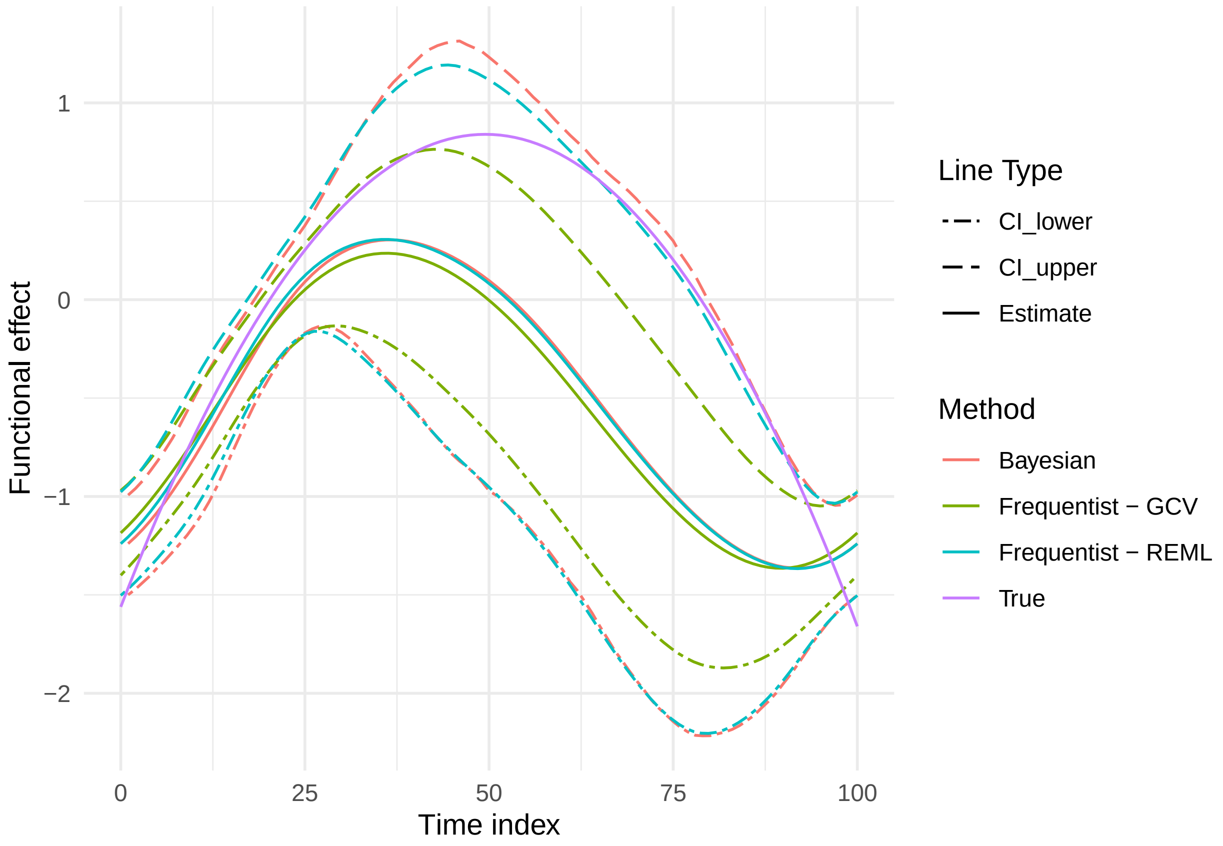

R code for fitting the equivalent model using refundBayes.

```r
refundBayes_SoFR = refundBayes::bfrs(y ~ X1+s(tmat, by=lmat*wmat, bs="cc", k=10),
      data = data.SoFR,
      family = binomial(),
      runStan = TRUE, # Whether automatically run Stan program.
      n.iter = 1500, # Total number of posterior sampling.
      n.warmup = 500, # Burn-in value.
      n.knots = 3 # Number of parallel computed chains for posterior sampling.
```

```r
    )

cat(refundBayes_SoFR$Stancode)
```

```
## data{
##    //Total number of observations
##    int<lower=1> N_num;
##    //Outcome variable
##    int Y[N_num];
##    real plocation;
##    real pscale;
##    //Number of scalar predictors
##    int<lower=1> K_num;
##    //Matrix of scalar predictors
##    matrix[N_num,K_num] Z_mat;
##    int<lower=1> Kr_1;
##    matrix[N_num, Kr_1] X_mat_r_1;
##    int<lower=1> Kf_1;
##    matrix[N_num, Kf_1] X_mat_f_1;
## }
## transformed data {
##    matrix[N_num, K_num] X_sc;
##    vector[K_num] mean_Xs;
##    for (i in 1:K_num) {
##       mean_Xs[i] = mean(Z_mat[, i]);
##      X_sc[, i] = Z_mat[, i] - mean_Xs[i];
##    }
## }
## parameters{
##    //Linear predictor intercept
##    real eta_0;
##    vector[K_num] gamma;
##    vector[Kr_1] zbr_1;
##    real<lower=0>sigmabr_1;
##    vector[Kf_1] bf_1;
## }
## transformed parameters {
##    real lprior = 0;
##    vector[Kr_1] br_1;
##    br_1 = sigmabr_1 * zbr_1;
## }
## model{
##    vector[N_num] mu = rep_vector(0.0, N_num);
##    //Linear predictor
##    mu += eta_0 + X_sc * gamma+ X_mat_r_1 * br_1+ X_mat_f_1 * bf_1;
##    for (n in 1:N_num) {
##      //Binomial log-likelihood
##      target += bernoulli_logit_lpmf(Y[n]|mu[n]);
##    }
##    target += student_t_lpdf(eta_0 | 3, plocation, pscale);
##    target += std_normal_lpdf(zbr_1);
##    target += inv_gamma_lpdf(sigmabr_1|0.0005,0.0005);
## }
```

```r
plot.bfrs(refundBayes_SoFR)
```

```
## [[1]]
```

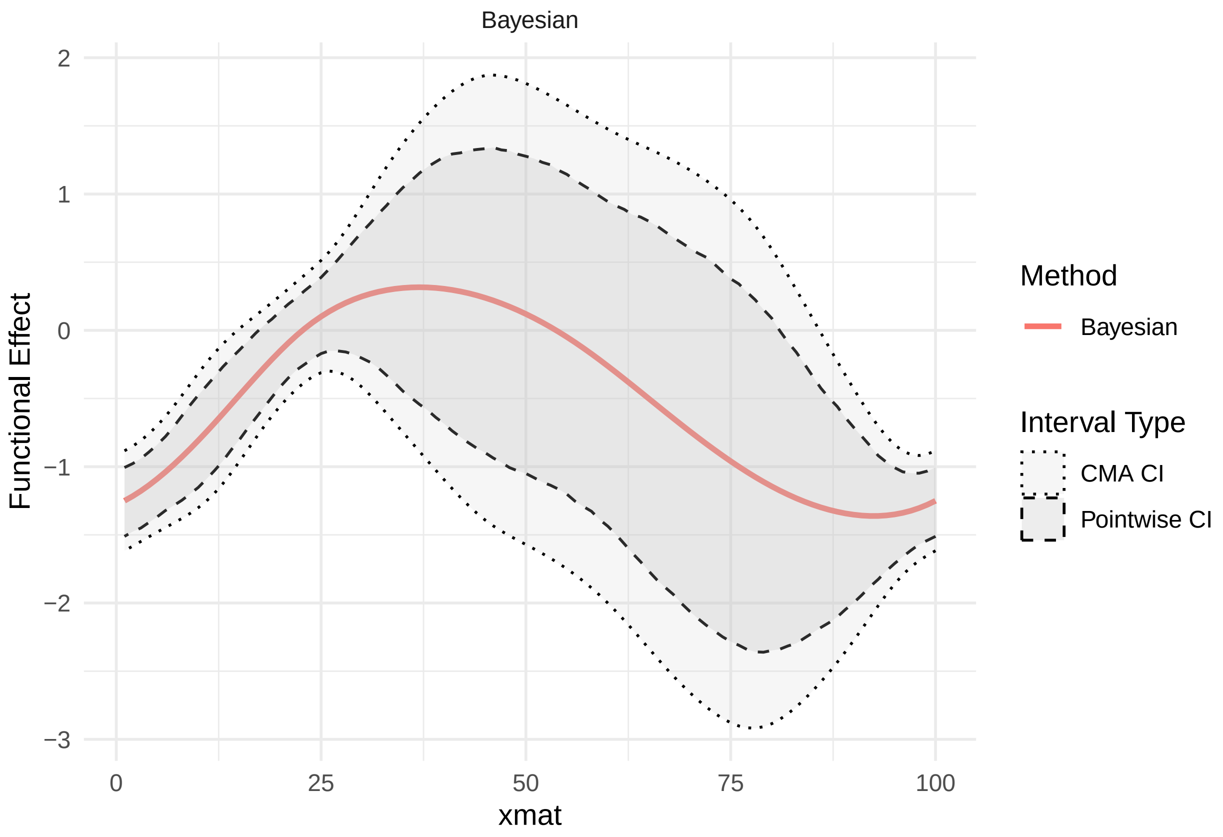

Section 3: Functional Cox regression

Stan code

```
stan_model_FunCox = stan_model(model_code = "
functions {
    real cox_log_lhaz(real y, real eta, real bhaz, real cbhaz)
        {return log(bhaz) + eta;}
    real cox_log_lccdf(real y, real eta, real bhaz, real cbhaz)
        {return - cbhaz * exp(eta);}
    real cox_log_lpdf(real y, real eta, real bhaz, real cbhaz)
        {return cox_log_lhaz(y, eta, bhaz, cbhaz) +cox_log_lccdf(y | eta, bhaz, cbhaz);}
}

data {
    int<lower=1> N_num;  // Total number of subjects
    real Y[N_num];  // Outcome variable
    int<lower=1> K_num;  // Number of scalar predictors
```

```
    // Design matrix for the scalar predictors
    matrix[K_num, N_num] Z_mat;
    int Kf; // Row number of the fixed effects design matrix
    int Kr; // Row number of the random effects design matrix
    matrix[Kf, N_num] X_mat_f; // Fixed effects design matrix
    matrix[Kr, N_num] X_mat_r; // Random effects design matrix
    array[N_num] int cens;
    int L_num;
    matrix[N_num, L_num] Mbasis;
    matrix[N_num, L_num] Ibasis;
}

parameters {
    real<lower=0> sigma; // Smoothing parameter
    real eta_0; // Linear predictor intercept
    vector[Kf] betaf; // Fixed effects spline coefficients
    vector[Kr] betar; // Random effects spline coefficients
    vector[K_num] gamma; // Coefficients for scalar predictors
    simplex[L_num] c;
}

model {
    // Construct the baseline hazard rate and cumulated baseline hazard rate
    vector[N_num] bhaz = Mbasis * c;
    vector[N_num] cbhaz = Ibasis * c;
    // Linear predictor
    vector[N_num] eta = rep_vector(0.0, N_num);
    eta += eta_0 + X_mat_f' * betaf + X_mat_r' * betar + Z_mat' * gamma;
    // Log-likelihood for the time-to-event outcome
    for (n in 1:N_num) {
        if (cens[n] == 0) {
            target += cox_log_lpdf(Y[n]| eta[n], bhaz[n], cbhaz[n]);
        }
        else if (cens[n] == 1) {
            target += cox_log_lccdf(Y[n]| eta[n], bhaz[n], cbhaz[n]);
        }
    }
    // Set priors for the parameters
    vector[L_num] alpha = rep_vector(1, L_num);
    target += dirichlet_lpdf(c | alpha);
    target += normal_lpdf(betar | 0, sigma);
    target += inv_gamma_lpdf(sigma^2 | 0.001, 0.001);
}")
```

Stan data

```
Func_Cox_Data = readRDS("data_func_Cox.rds")
Func_Cox_Data$wmat = Func_Cox_Data$MIMS

y.use = Func_Cox_Data$survtime
```

```r
smcon = mgcv:::smoothCon(s(tmat, by=lmat*wmat, bs="cc", k=10),data = Func_Cox_Data,absorb.cons = TRUE,di
randeff = mgcv::smooth2random(smcon[[1]], names(Func_Cox_Data), type = 2)
X_mat_r = t(randeff$rand$Xr)
X_mat_f = t(randeff$Xf)
Mbasis = splines2::mSpline(x = y.use, Boundary.knots=c(54, 123), df=5, intercept=TRUE)
Ibasis = splines2::iSpline(x = y.use, Boundary.knots=c(54, 123), df=5)

# Stan data list
data_stan = list()
data_stan[["N_num"]] = length(y.use)
data_stan[["Y"]] = c(y.use)
data_stan[["K_num"]] = 1
data_stan[["Z_mat"]] = (t(Func_Cox_Data$X1))
data_stan[["Kr"]]= NROW(X_mat_r)
data_stan[["Kf"]]= NROW(X_mat_f)
data_stan[["X_mat_r"]] = X_mat_r
data_stan[["X_mat_f"]] = X_mat_f
data_stan[["cens"]] = 1-Func_Cox_Data$event
data_stan[["L_num"]] = 5
data_stan[["Mbasis"]] = Mbasis
data_stan[["Ibasis"]] = Ibasis



Bayes_fit_FunCox = sampling(stan_model_FunCox, data = data_stan, iter = 5000,warmup = 2000,chain=1,core
```

```
##
## SAMPLING FOR MODEL 'anon_model' NOW (CHAIN 1).
## Chain 1:
## Chain 1: Gradient evaluation took 0.000138 seconds
## Chain 1: 1000 transitions using 10 leapfrog steps per transition would take 1.38 seconds.
## Chain 1: Adjust your expectations accordingly!
## Chain 1:
## Chain 1:
## Chain 1: Iteration:    1 / 5000 [  0%]  (Warmup)
## Chain 1: Iteration:  500 / 5000 [ 10%]  (Warmup)
## Chain 1: Iteration: 1000 / 5000 [ 20%]  (Warmup)
## Chain 1: Iteration: 1500 / 5000 [ 30%]  (Warmup)
## Chain 1: Iteration: 2000 / 5000 [ 40%]  (Warmup)
## Chain 1: Iteration: 2001 / 5000 [ 40%]  (Sampling)
## Chain 1: Iteration: 2500 / 5000 [ 50%]  (Sampling)
## Chain 1: Iteration: 3000 / 5000 [ 60%]  (Sampling)
## Chain 1: Iteration: 3500 / 5000 [ 70%]  (Sampling)
## Chain 1: Iteration: 4000 / 5000 [ 80%]  (Sampling)
## Chain 1: Iteration: 4500 / 5000 [ 90%]  (Sampling)
## Chain 1: Iteration: 5000 / 5000 [100%]  (Sampling)
## Chain 1:
## Chain 1:  Elapsed Time: 2.168 seconds (Warm-up)
## Chain 1:                2.625 seconds (Sampling)
## Chain 1:                4.793 seconds (Total)
## Chain 1:
```

Construct the estimated functional coefficient

```
object = s(tmat, by=lmat*wmat, bs="cc", k=10)
data = Func_Cox_Data
knots = NULL
dk = ExtractData(object,data,knots)
splinecons = smooth.construct.cc.smooth.spec(object,dk$data,dk$knots)

Psi_mat = splinecons$X
S_mat = splinecons$S[[1]]
rank = splinecons$rank


##############################################################################
###     Calculate the X_mat, following the internal code of mgcv
##############################################################################

bymat=Func_Cox_Data$lmat*Func_Cox_Data$wmat
Psi_mat.new=matrix(1,nrow = prod(dim(bymat)), ncol=NCOL(Psi_mat))
for(i in 1:NROW(Psi_mat)){
  for(inx in ((i-1)*dim(bymat)[1]+1):(i*dim(bymat)[1])){
    Psi_mat.new[inx,]=Psi_mat[i,]
  }
}
Psi_mat = Psi_mat.new
maXX = norm(Psi_mat,type="I")^2
maS = norm(S_mat)/maXX
S_mat = S_mat / maS
eigendecomp = eigen(S_mat, symmetric = TRUE)
U_mat = eigendecomp$vectors
V_vec = eigendecomp$value[1:rank]

smlX <- as.numeric(bymat)*Psi_mat
n_num = NROW(Func_Cox_Data)
ind <- 1:n_num
X_mat <- smlX[ind,,drop=FALSE]
for (i in 2:dim(data$wmat)[2]) {
  ind <- ind + n_num
  X_mat <- X_mat +smlX[ind,,drop=FALSE]
}
X_mat = X_mat %*% U_mat

##############################################################################
###     Reconstruct the spline coefficients 'beta.sample.untilde'
##############################################################################

V = rep(1, ncol(X_mat))
V[1:rank] = sqrt(V_vec)
col.norm = colSums((X_mat)^2)
col.norm = col.norm / V^2
av.norm = mean(col.norm[1:rank])
for (i in (rank + 1):ncol(X_mat)) {
  V[i] = sqrt(col.norm[i] / av.norm)
```

```r
}

post_sample = rstan::extract(Bayes_fit_FunCox)
beta.sample = t(cbind(post_sample$betar,post_sample$betaf) %*% randeff$trans.U)
beta.sample.untilde = (U_mat %*% diag(1 / V)) %*% beta.sample


##############################################################################
###     Reconstruct the estimated functional coefficient
##############################################################################

beta.post = apply(beta.sample.untilde, 2, function(x){x%*%t(splinecons$X)})

mean.curve.est=apply(beta.post,1,mean)
upper.curve.est=apply(beta.post,1,function(x){quantile(x,probs = 0.975)})
lower.curve.est=apply(beta.post,1,function(x){quantile(x,probs = 0.025)})

plotdata=data.frame(value=c(mean.curve.est,
                            upper.curve.est,
                            lower.curve.est),
                    xmat=c(rep(1: dim(Func_Cox_Data$wmat)[2],3)),
                    Method=c(rep("Bayesian",dim(Func_Cox_Data$wmat)[2]),
                           rep("Bayesian",dim(Func_Cox_Data$wmat)[2]),
                           rep("Bayesian",dim(Func_Cox_Data$wmat)[2])),
                    type=c(rep("Estimate",dim(Func_Cox_Data$wmat)[2]),
                           rep("CI_upper",dim(Func_Cox_Data$wmat)[2]),
                           rep("CI_lower",dim(Func_Cox_Data$wmat)[2])))

library(ggplot2)
ggplot(plotdata,aes(y=value,x=xmat))+geom_line(aes(linetype=type,color=Method))+
  ylab("Functional effect")+xlab("Time (hour)")+
  #scale_x_continuous(breaks=seq(0,1,by=3))+
  scale_linetype_manual(values=c("twodash", "longdash","solid"),name="Line Type")+
  theme_minimal()
```

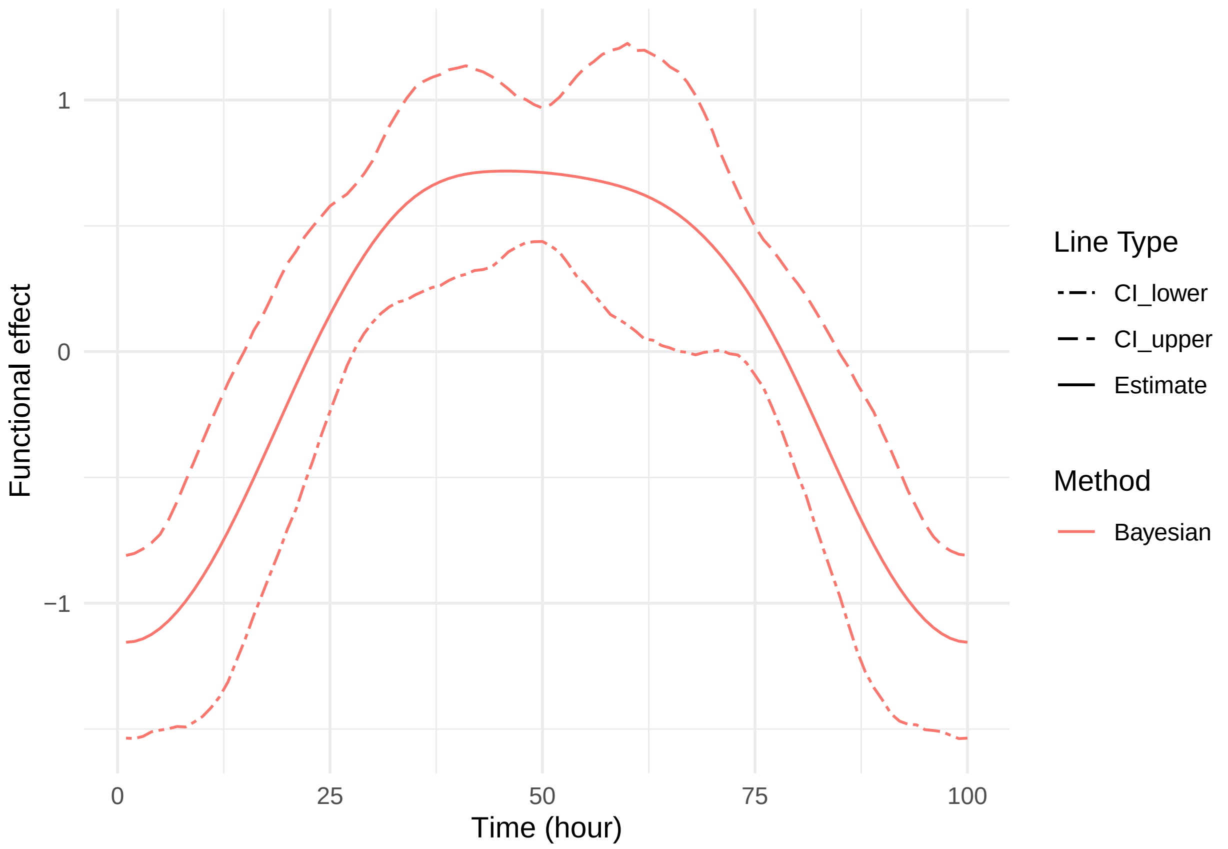

Compare with frequentist mgcv result

```
fit_freq1<- gam(survtime ~ s(tmat, by=lmat*wmat, bs="cc", k=10)+X1, weights=event, data=Func_Cox_Data, 
plotfot=plot.gam(fit_freq1,unconditional = FALSE)
```

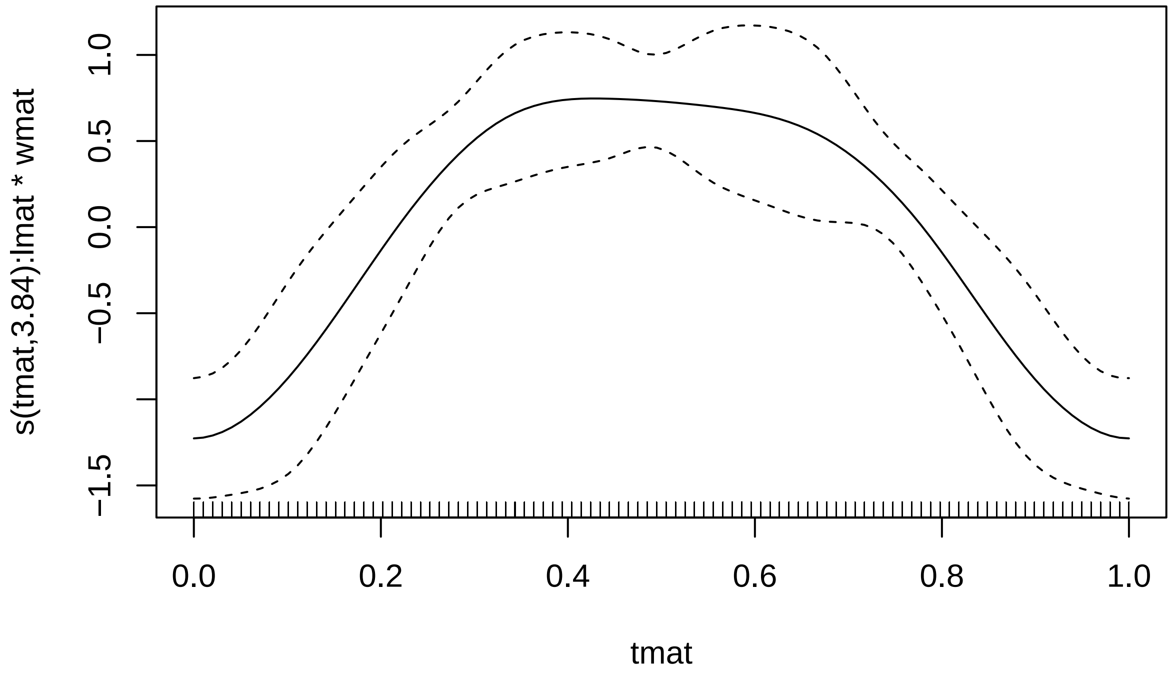


```
plotdata=data.frame(value=c(mean.curve.est,
                            upper.curve.est,
                            lower.curve.est,
                            plotfot[[1]]$fit,
                            plotfot[[1]]$fit+plotfot[[1]]$se,
                            plotfot[[1]]$fit-plotfot[[1]]$se),
                    xmat=c(rep(1: T_num,3),
                           rep(plotfot[[1]]$x*100,3)),
                    Method=c(rep("Bayesian",T_num),
                           rep("Bayesian",T_num),
                           rep("Bayesian",T_num),
                           rep("Frequentist",100),
                           rep("Frequentist",100),
                           rep("Frequentist",100)),
                    type=c(rep("Estimate",T_num),
                           rep("CI_upper",T_num),
                           rep("CI_lower",T_num),
                           rep("Estimate",100),
                           rep("CI_upper",100),
                           rep("CI_lower",100)))

library(ggplot2)
ggplot(plotdata,aes(y=value,x=xmat))+geom_line(aes(linetype=type,color=Method))+
  ylab("Functional effect")+xlab("Time (hour)")+
  #scale_x_continuous(breaks=seq(0,1,by=3))+
  scale_linetype_manual(values=c("twodash", "longdash","solid"),name="Line Type")+
```

```r
  theme_minimal()
```

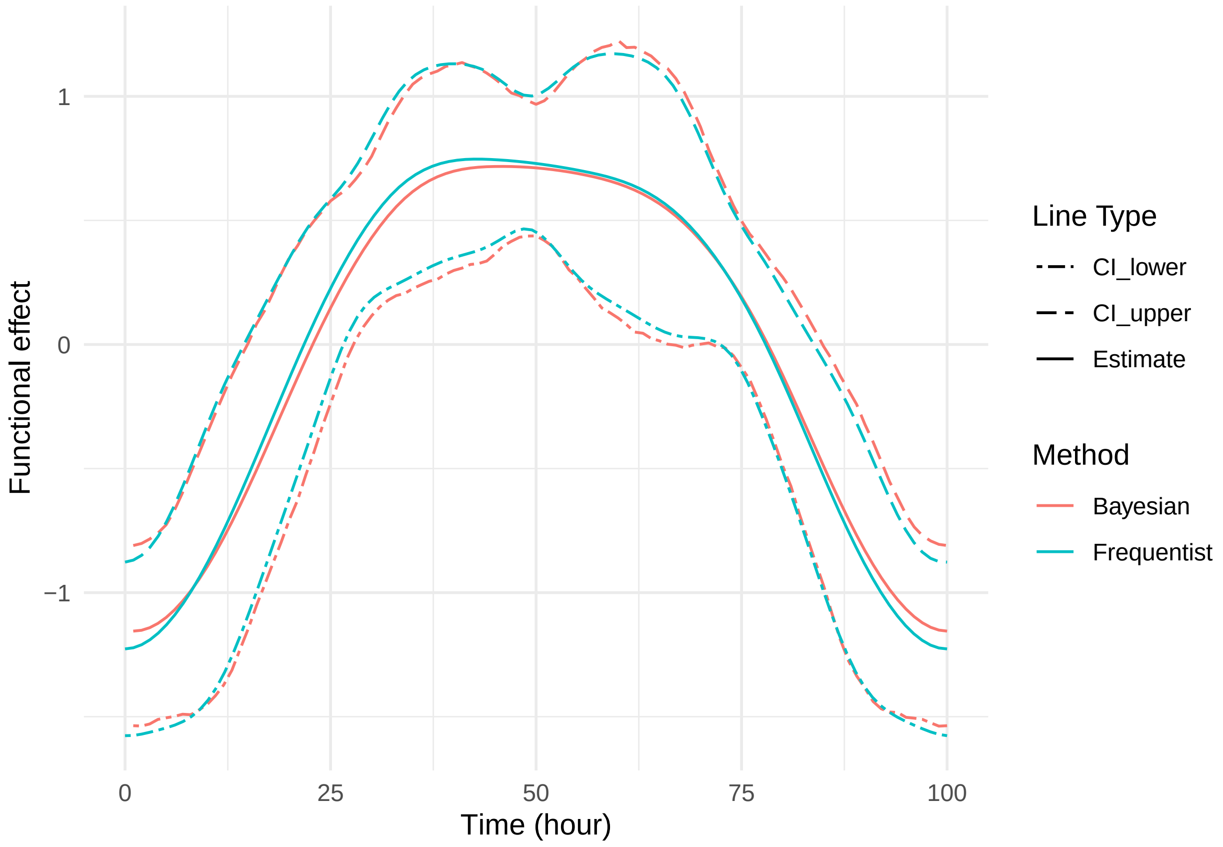

R code for fitting the equivalent model using refundBayes.

```r
# Censoring equals 1 - event
Func_Cox_Data$cens = 1 - Func_Cox_Data$event

refundBayes_FunCox = refundBayes::bfrs(survtime ~ X1+s(tmat, by=lmat*wmat, bs="cc", k=10),
      data = Func_Cox_Data,
      family = "Cox",
      cens = "cens",
      runStan = TRUE, # Whether automatically run Stan program.
      n.iter = 5000, # Total number of posterior sampling.
      n.warmup = 2000, # Burn-in value.
      n.knots = 3 # Number of parallel computed chains for posterior sampling.
      )
```

```
## Warning: There were 100 divergent transitions after warmup. See
## https://mc-stan.org/misc/warnings.html#divergent-transitions-after-warmup
## to find out why this is a problem and how to eliminate them.

## Warning: Examine the pairs() plot to diagnose sampling problems
```

```r
cat(refundBayes_FunCox$Stancode)
```

```
## functions {
##    real cox_log_lhaz(real y, real log_mu, real bhaz, real cbhaz) {
##       return log(bhaz) + log_mu;
##    }
##    real cox_log_lccdf(real y, real log_mu, real bhaz, real cbhaz) {
##       return - cbhaz * exp(log_mu);
##    }
##    real cox_log_lcdf(real y, real log_mu, real bhaz, real cbhaz) {
##       return log1m_exp(cox_log_lccdf(y | log_mu, bhaz, cbhaz));
##    }
##    real cox_log_lpdf(real y, real log_mu, real bhaz, real cbhaz) {
##       return cox_log_lhaz(y, log_mu, bhaz, cbhaz) +
##              cox_log_lccdf(y | log_mu, bhaz, cbhaz);
##    }
## }
## data{
##    //Total number of observations
##    int<lower=1> N_num;
##    real real_inter;
##    //Indicator variable for censoring
##    array[N_num] int<lower=-1,upper=2> cens;
##    //Number of baseline hazard function basis
##    int L_num;
##    vector<lower=0>[L_num] con_sbhaz;
##    //Baseline hazard function basis
##    matrix[N_num, L_num] Mbasis;
##    //Cumulative baseline hazard function basis
##    matrix[N_num, L_num] Ibasis;
##    //Time-to-event outcome variable
##    real Y[N_num];
##    //Number of scalar predictors
##    int<lower=1> K_num;
##    //Matrix of scalar predictors
##    matrix[N_num,K_num] Z_mat;
##    int<lower=1> Kr_1;
##    matrix[N_num, Kr_1] X_mat_r_1;
##    int<lower=1> Kf_1;
##    matrix[N_num, Kf_1] X_mat_f_1;
## }
## transformed data {
##    matrix[N_num, K_num] X_sc;
##    vector[K_num] mean_Xs;
##    for (i in 1:K_num) {
##       mean_Xs[i] = mean(Z_mat[, i]);
##      X_sc[, i] = Z_mat[, i] - mean_Xs[i];
##    }
## }
## parameters{
##    //Linear predictor intercept
##    real eta_0;
##    //Baseline hazard spline coefficient
```

```
##    simplex[L_num] c;
##    vector[K_num] gamma;
##    vector[Kr_1] zbr_1;
##    real<lower=0>sigmabr_1;
##    vector[Kf_1] bf_1;
## }
## transformed parameters {
##    real lprior = 0;
##    vector[Kr_1] br_1;
##    br_1 = sigmabr_1 * zbr_1;
## }
## model{
##    vector[N_num] mu = rep_vector(0.0, N_num);
##    //Linear predictor
##    mu += eta_0 + X_sc * gamma+ X_mat_r_1 * br_1+ X_mat_f_1 * bf_1;
##    //Fit the baseline hazard function
##    vector[N_num] bhaz = Mbasis * c;
##    //Fit the cumulative baseline hazard function
##    vector[N_num] cbhaz = Ibasis * c;
##    for (n in 1:N_num) {
##     if (cens[n] == 0) {
##       target += cox_log_lpdf(Y[n] | mu[n], bhaz[n], cbhaz[n]);
##     } else if (cens[n] == 1) {;
##       target += cox_log_lccdf(Y[n] | mu[n], bhaz[n], cbhaz[n]);
##     } else if (cens[n] == -1) {
##       target += cox_log_lcdf(Y[n] | mu[n], bhaz[n], cbhaz[n]);
##     }
##    }
##    target += normal_lpdf(eta_0 | real_inter, 10);
##    target += dirichlet_lpdf(c | con_sbhaz);
##    target += std_normal_lpdf(zbr_1);
##    target += inv_gamma_lpdf(sigmabr_1|0.0005,0.0005);
## }
```

```
plot.bfrs(refundBayes_FunCox)
```

```
## [[1]]
```

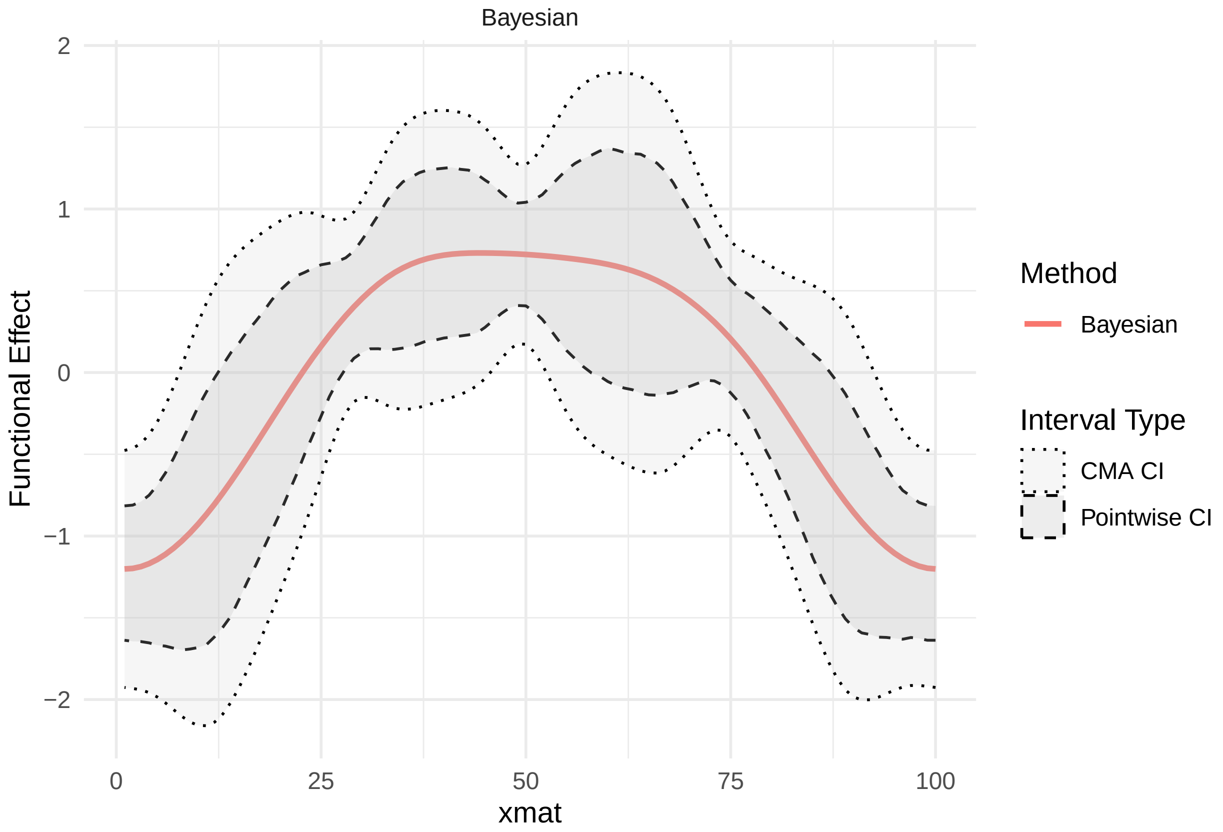

Section 4: Bayesian Joint Functional Models with FPCA

Stan code

```
stan_model_jointFPCA = stan_model(model_code = "
functions {
    real cox_log_lhaz(real y, real eta, real bhaz, real cbhaz)
        {return log(bhaz) + eta;}
    real cox_log_lccdf(real y, real eta, real bhaz, real cbhaz)
        {return - cbhaz * exp(eta);}
    real cox_log_lpdf(real y, real eta, real bhaz, real cbhaz)
        {return cox_log_lhaz(y, eta, bhaz, cbhaz) +cox_log_lccdf(y | eta, bhaz, cbhaz);}
}

data {
    int<lower=1> N_num;  // Total number of subjects
    int<lower=1> T_num;  // Total number of subjects
    matrix[N_num, T_num] M_mat;

    real Y[N_num];  // Outcome variable
    int<lower=1> K_num;  // Number of scalar predictors
    matrix[K_num, N_num] Z_mat;
```

```
    int Kf; // Row number of the fixed effects design matrix
    int Kr; // Row number of the random effects design matrix
    int J_num; //
    int L_num;
    int M_num;
    matrix[Kf, J_num] X_mat_f; // Fixed effects design matrix
    matrix[Kr, J_num] X_mat_r; // Random effects design matrix
    array[N_num] int cens;
    matrix[N_num, L_num] Mbasis;
    matrix[N_num, L_num] Ibasis;


    matrix[J_num,T_num] Phi_mat;  // estimated level 1 eigenfunction
    matrix[N_num,J_num] xi_hat;

}

parameters {
    real<lower=0> sigma; // Smoothing parameter
    real eta_0; // Linear predictor intercept
    vector[Kf] betaf; // Fixed effects spline coefficients
    vector[Kr] betar; // Random effects spline coefficients
    vector[K_num] gamma; // Coefficients for scalar predictors
    simplex[L_num] c;
    matrix[N_num, J_num] xi;
    real<lower = 0> sigma_e;
    real<lower=0> lambda[J_num];
}

model {
    // Baseline and cumulative baseline hazard functions
    vector[N_num] bhaz = Mbasis * c;
    vector[N_num] cbhaz = Ibasis * c;
    // Linear predictor
    vector[N_num] eta = rep_vector(0.0, N_num);
    eta += eta_0 + xi * X_mat_f' * betaf + xi * X_mat_r' * betar + Z_mat' * gamma;
    // Likelihood for the time-to-event outcome
    for (n in 1:N_num) {
        if (cens[n] == 0) {
            target += cox_log_lpdf(Y[n] | eta[n], bhaz[n], cbhaz[n]);
        }
        else if (cens[n] == 1) {
            target += cox_log_lccdf(Y[n] | eta[n], bhaz[n], cbhaz[n]);
        }
    }
    // Likelihood for the functional observation in FPCA
    target += - N_num * M_num * log(sigma_e) - sum((xi * Phi_mat - M_mat)^2) / (2 * sigma_e^2);
    // Set prior for the FPCA scores xi
    for(nj in 1:J_num){
        target += - N_num * log(lambda[nj]) -
        sum((xi[,nj] - xi_hat[,nj])^2) / (2 * lambda[nj]^2);
    }
    // Other priors
```

```
    vector[L_num] alpha = rep_vector(1, L_num);
    target += dirichlet_lpdf(c | alpha);
    target += normal_lpdf(betar | 0, sigma);
    target += inv_gamma_lpdf(sigma^2|0.001,0.001);
    for(nj in 1:J_num){
        target += inv_gamma_lpdf(lambda[nj]|0.001,0.001);
    }
    target += inv_gamma_lpdf(sigma_e^2|0.001,0.001);
}")
```

Stan data

```
data = Func_Cox_Data
fpca.fit = refund::fpca.sc(Y=data$wmat)

object = s(tmat, by=lmat*wmat, bs="cc", k=10)
dk = ExtractData(object,data,knots)
splinecons = smooth.construct.cc.smooth.spec(object,dk$data,dk$knots)

Psi_mat = splinecons$X
S_mat = splinecons$S[[1]]

M_num = dim(splinecons$X)[1]
J_num = dim(fpca.fit$efunctions)[2]
K_num = dim(splinecons$X)[2]

X_mat_t = matrix(nrow=J_num, ncol= K_num)
for(j in 1:J_num){
  for(k in 1:K_num) {
    X_mat_t[j,k] = sum(fpca.fit$efunctions[,j] * Psi_mat[,k]) / M_num
  }
}


maXX = norm(Psi_mat,type="I")^2
maS = norm(S_mat)/maXX
S_mat = S_mat / maS
eigendecomp=eigen(S_mat, symmetric = TRUE)

rank=splinecons$rank

E = rep(1, ncol(X_mat_t))
E[1:rank] = sqrt(eigendecomp$value[1:rank])
X_mat_t = X_mat_t %*% eigendecomp$vectors
col.norm = colSums(X_mat_t^2)
col.norm = col.norm/E^2
av.norm = mean(col.norm[1:rank])
for (i in (rank + 1):ncol(X_mat_t)) {
  E[i] = sqrt(col.norm[i]/av.norm)
}

X_mat_t = t(t(X_mat_t)/E)
```

```
X_mat_r = t(X_mat_t[,1:rank])
X_mat_f = t(X_mat_t[,(rank + 1):ncol(X_mat_t)])



Func_Cox_Data = readRDS("data_func_Cox.rds")
Func_Cox_Data$wmat = Func_Cox_Data$MIMS

y.use = Func_Cox_Data$survtime

smcon = mgcv:::smoothCon(s(tmat, by=lmat*wmat, bs="cc", k=10),data = Func_Cox_Data,absorb.cons = TRUE,d[illegible]
randeff = mgcv::smooth2random(smcon[[1]], names(Func_Cox_Data), type = 2)

Mbasis = splines2::mSpline(x = y.use, Boundary.knots=c(54, 123), df=5, intercept=TRUE)
Ibasis = splines2::iSpline(x = y.use, Boundary.knots=c(54, 123), df=5)

# Stan data list
data_stan = list()
data_stan[["N_num"]] = length(y.use)
data_stan[["Y"]] = c(y.use)
data_stan[["K_num"]] = 1
data_stan[["Z_mat"]] = (t(Func_Cox_Data$X1))
data_stan[["Kr"]]= NROW(X_mat_r)
data_stan[["Kf"]]= NROW(X_mat_f)
data_stan[["X_mat_r"]] = X_mat_r
data_stan[["X_mat_f"]] = X_mat_f
data_stan[["cens"]] = 1-Func_Cox_Data$event
data_stan[["L_num"]] = 5
data_stan[["Mbasis"]] = Mbasis
data_stan[["Ibasis"]] = Ibasis

data_stan[["J_num"]] = J_num
data_stan[["M_num"]] = M_num
data_stan[["Phi_mat"]] = t(fpca.fit$efunctions)
data_stan[["xi_hat"]] = fpca.fit$scores
data_stan[["T_num"]] = T_num
data_stan[["M_mat"]] = data$wmat


Bayes_fit_jointFPCA = sampling(stan_model_jointFPCA, data = data_stan, iter = 500,warmup = 200,chain=1,[illegible]
```

```
##
## SAMPLING FOR MODEL 'anon_model' NOW (CHAIN 1).
## Chain 1:
## Chain 1: Gradient evaluation took 0.001882 seconds
## Chain 1: 1000 transitions using 10 leapfrog steps per transition would take 18.82 seconds.
## Chain 1: Adjust your expectations accordingly!
## Chain 1:
## Chain 1:
## Chain 1: Iteration:   1 / 500 [  0%]  (Warmup)
## Chain 1: Iteration:  50 / 500 [ 10%]  (Warmup)
## Chain 1: Iteration: 100 / 500 [ 20%]  (Warmup)
## Chain 1: Iteration: 150 / 500 [ 30%]  (Warmup)
```

```
## Chain 1: Iteration: 200 / 500 [ 40%]  (Warmup)
## Chain 1: Iteration: 201 / 500 [ 40%]  (Sampling)
## Chain 1: Iteration: 250 / 500 [ 50%]  (Sampling)
## Chain 1: Iteration: 300 / 500 [ 60%]  (Sampling)
## Chain 1: Iteration: 350 / 500 [ 70%]  (Sampling)
## Chain 1: Iteration: 400 / 500 [ 80%]  (Sampling)
## Chain 1: Iteration: 450 / 500 [ 90%]  (Sampling)
## Chain 1: Iteration: 500 / 500 [100%]  (Sampling)
## Chain 1:
## Chain 1:  Elapsed Time: 11.581 seconds (Warm-up)
## Chain 1:                9.991 seconds (Sampling)
## Chain 1:                21.572 seconds (Total)
## Chain 1:

## Warning: There were 1 chains where the estimated Bayesian Fraction of Missing Information was low. Se
## https://mc-stan.org/misc/warnings.html#bfmi-low

## Warning: Examine the pairs() plot to diagnose sampling problems

## Warning: The largest R-hat is 1.84, indicating chains have not mixed.
## Running the chains for more iterations may help. See
## https://mc-stan.org/misc/warnings.html#r-hat

## Warning: Bulk Effective Samples Size (ESS) is too low, indicating posterior means and medians may be
## Running the chains for more iterations may help. See
## https://mc-stan.org/misc/warnings.html#bulk-ess

## Warning: Tail Effective Samples Size (ESS) is too low, indicating posterior variances and tail quant
## Running the chains for more iterations may help. See
## https://mc-stan.org/misc/warnings.html#tail-ess
```

Plot the functional coefficient

```
post_sample = rstan::extract(Bayes_fit_jointFPCA)
beta.sample = t(cbind(post_sample$betar,post_sample$betaf))
beta.sample.untilde = (eigendecomp$vectors %*% diag(1 / E)) %*% beta.sample


##############################################################################
###     Reconstruct the estimated functional coefficient
##############################################################################

beta.post = apply(beta.sample.untilde, 2, function(x){x%*%t(splinecons$X)})

mean.curve.est=apply(beta.post,1,mean)
upper.curve.est=apply(beta.post,1,function(x){quantile(x,probs = 0.975)})
lower.curve.est=apply(beta.post,1,function(x){quantile(x,probs = 0.025)})

plotdata=data.frame(value=c(mean.curve.est,
                            upper.curve.est,
                            lower.curve.est),
```

```
                    xmat=c(rep(1: dim(Func_Cox_Data$wmat)[2],3)),
                    Method=c(rep("Bayesian",dim(Func_Cox_Data$wmat)[2]),
                           rep("Bayesian",dim(Func_Cox_Data$wmat)[2]),
                           rep("Bayesian",dim(Func_Cox_Data$wmat)[2])),
                    type=c(rep("Estimate",dim(Func_Cox_Data$wmat)[2]),
                           rep("CI_upper",dim(Func_Cox_Data$wmat)[2]),
                           rep("CI_lower",dim(Func_Cox_Data$wmat)[2])))

library(ggplot2)
ggplot(plotdata,aes(y=value,x=xmat))+geom_line(aes(linetype=type,color=Method))+
  ylab("Functional effect")+xlab("Time (hour)")+
  #scale_x_continuous(breaks=seq(0,1,by=3))+
  scale_linetype_manual(values=c("twodash", "longdash","solid"),name="Line Type")+
  theme_minimal()
```

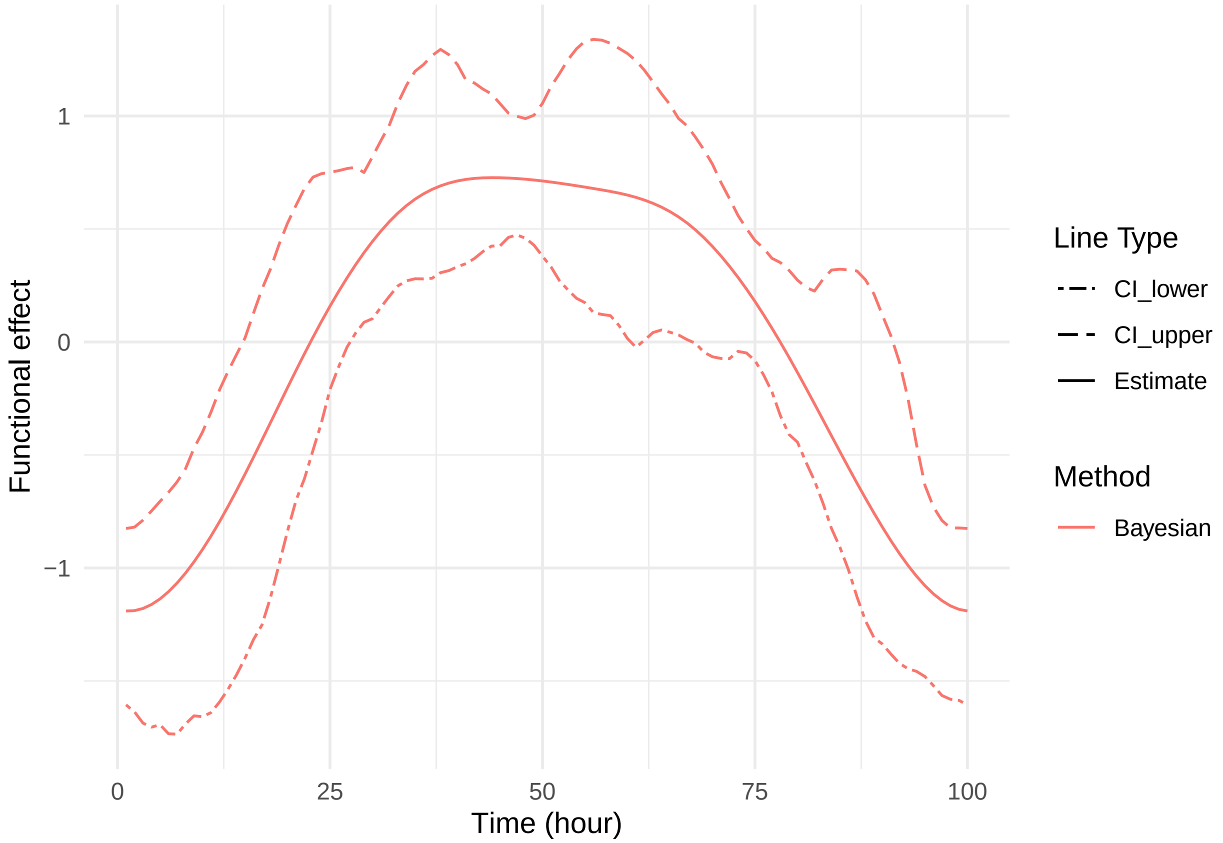

Section 5: Bayesian function-on-scalar regression

Stan data

```
stan_model_FoSR = stan_model(model_code = "
data {
    int<lower=1> N_num; // Total number of participants
```

```
    int<lower=1> M_num; // Total number of observed functional time point
    matrix[N_num, M_num] Y_mat; // Functional response
    int<lower=1> P_num; // Number of scalar predictors
    matrix[N_num, P_num] X_mat; // Design matrix for the scalar predictor
    int<lower=1> J_num; // Number of FPCA eigenfunctions
    matrix[J_num, M_num] Phi_mat; // Matrix of FPCA eigenfunctions
    int<lower=1> K_num; // Number of spline basis
    matrix[K_num, M_num] Psi_mat; // Matrix of spline basis
    matrix[K_num, K_num] S_mat; // Penalty matrix
}

parameters {
    matrix[K_num, P_num] beta; // Spline coefficients
    matrix[N_num, J_num] xi; // FPCA Scores
    real<lower=0> sigma_eps; // Standard deviation of independent error
    vector<lower=0>[P_num] sigma; // Smoothing parameter
    vector<lower=0>[J_num] lambda; // FPCA eigenvalues
}

model {
    matrix[N_num, M_num] mu;
    // Fitted mean matrix
    mu = X_mat * beta' * Psi_mat + xi * Phi_mat;
    // Log-likelihood for functional response
    target += - N_num * M_num * log(sigma_eps) / 2 - sum((mu - Y_mat)^2) / (2 * sigma_eps^2);
    // Prior for the penalized spline coefficients
    for(np in 1:P_num){
        target += (- beta[,np]' * S_mat * beta[,np]) / (2 * sigma[np]^2);
        target += inv_gamma_lpdf(sigma[np]^2|0.001,0.001);
    }
    // Prior for the FPCA scores
    for(nj in 1:J_num){
        target += - N_num * log(lambda[nj]) / 2 - sum((xi[,nj])^2) / (2 * lambda[nj]^2);
        target += inv_gamma_lpdf(lambda[nj]^2|0.001,0.001);
    }
    // Other priors
    target += inv_gamma_lpdf(sigma_eps^2|0.001,0.001);
}
generated quantities {
    matrix[P_num, M_num] beta_est = beta' * Psi_mat;
}")
```

Frequentist method

```
FoSR_exp_data = readRDS("FoSR_exp_data.rds")
FoSR_exp_data$y = FoSR_exp_data$MIMS

fit.freq = pffr(y~-1+X,data=FoSR_exp_data,bs.yindex=list(bs="cc", k=10))
plotfot = plot(fit.freq)
```

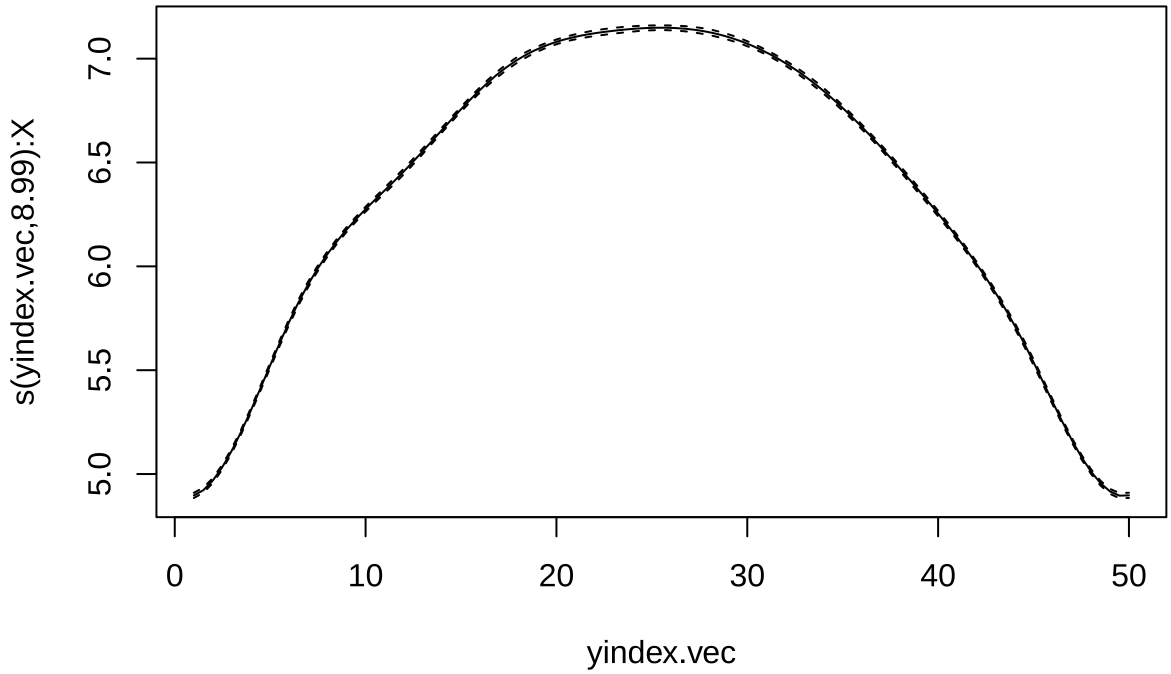

Stan data

```
T_num  = 50
n_num = 300
tind = seq(0, 1, length.out = T_num)

FoSR_exp_data[["lmat"]] = I(matrix(1/T_num, ncol = T_num, nrow = n_num))
FoSR_exp_data[["tmat"]] = I(matrix(tind, ncol = T_num, nrow = n_num, byrow = TRUE))

fpca.fit = refund::fpca.face(unclass(FoSR_exp_data$y))

FoSR_exp_data[["yindex.vec"]]=matrix(fit.freq[["model"]][["yindex.vec"]],nrow = n_num)
object=s(yindex.vec,  bs = "cc", k = 10)
data = FoSR_exp_data
knots = NULL
dk = ExtractData(object,data,knots)

splinecons = smooth.construct.cc.smooth.spec(object,dk$data,dk$knots)
Psi_mat=splinecons$X
S_mat=splinecons$S[[1]]


maXX = norm(Psi_mat,type="I")^2
maS = norm(S_mat)/maXX
```

```
S_mat = S_mat / maS

data_stan=list()
data_stan[["Y_mat"]] = FoSR_exp_data$y
data_stan[["N_num"]] = dim(FoSR_exp_data$y)[1]
data_stan[["M_num"]] = dim(FoSR_exp_data$y)[2]
data_stan[["P_num"]] = 1
data_stan[["Phi_mat"]] = t(fpca.fit$efunctions)
data_stan[["K_num"]] = dim(Psi_mat)[2]
data_stan[["X_mat"]] = matrix(FoSR_exp_data$X, ncol = 1)
data_stan[["Psi_mat"]] = t(Psi_mat)
data_stan[["J_num"]] = ifelse(is.null(dim(fpca.fit$efunctions)[2]),1,dim(fpca.fit$efunctions)[2])
data_stan[["S_mat"]] = S_mat

#data_stan[["sigma_k"]]=fpca.fit$evalues


Bayes_fit_FoSR = sampling(stan_model_FoSR, data = data_stan, iter = 2000,warmup = 1000,chain=3,cores = [illegible]
```

Compare with frequentist mgcv result

```
post_sample = rstan::extract(Bayes_fit_FoSR)
#beta.sample = t(post_sample$beta[,,1])

beta.post = t(post_sample$beta_est[,1,])
mean.curve.est=apply(beta.post,1,mean)
upper.curve.est=apply(beta.post,1,function(x){quantile(x,probs = 0.975)})
lower.curve.est=apply(beta.post,1,function(x){quantile(x,probs = 0.025)})

plotdata=data.frame(value=c(mean.curve.est,
                            upper.curve.est,
                            lower.curve.est),
                    xmat=c(rep(1: dim(FoSR_exp_data$y)[2],3)),
                    Method=c(rep("Bayesian",dim(FoSR_exp_data$y)[2]),
                           rep("Bayesian",dim(FoSR_exp_data$y)[2]),
                           rep("Bayesian",dim(FoSR_exp_data$y)[2])),
                    type=c(rep("Estimate",dim(FoSR_exp_data$y)[2]),
                           rep("CI_upper",dim(FoSR_exp_data$y)[2]),
                           rep("CI_lower",dim(FoSR_exp_data$y)[2])))


plotdata=data.frame(value=c(mean.curve.est,
                            upper.curve.est,
                            lower.curve.est,
                            plotfot[[1]]$fit,
                            plotfot[[1]]$fit+plotfot[[1]]$se,
                            plotfot[[1]]$fit-plotfot[[1]]$se),
                    xmat=c(rep(1: T_num,3),
                           rep(plotfot[[1]]$x,3)),
                    Method=c(rep("Bayesian",T_num),
                           rep("Bayesian",T_num),
```

```
                        rep("Bayesian",T_num),
                        rep("Frequentist",100),
                        rep("Frequentist",100),
                        rep("Frequentist",100)),
                type=c(rep("Estimate",T_num),
                        rep("CI_upper",T_num),
                        rep("CI_lower",T_num),
                        rep("Estimate",100),
                        rep("CI_upper",100),
                        rep("CI_lower",100)))


library(ggplot2)
ggplot(plotdata,aes(y=value,x=xmat))+geom_line(aes(linetype=type,color=Method))+
  ylab("Functional effect")+xlab("Time (hour)")+
  #scale_x_continuous(breaks=seq(0,1,by=3))+
  scale_linetype_manual(values=c("twodash", "longdash","solid"),name="Line Type")+
  theme_minimal()
```

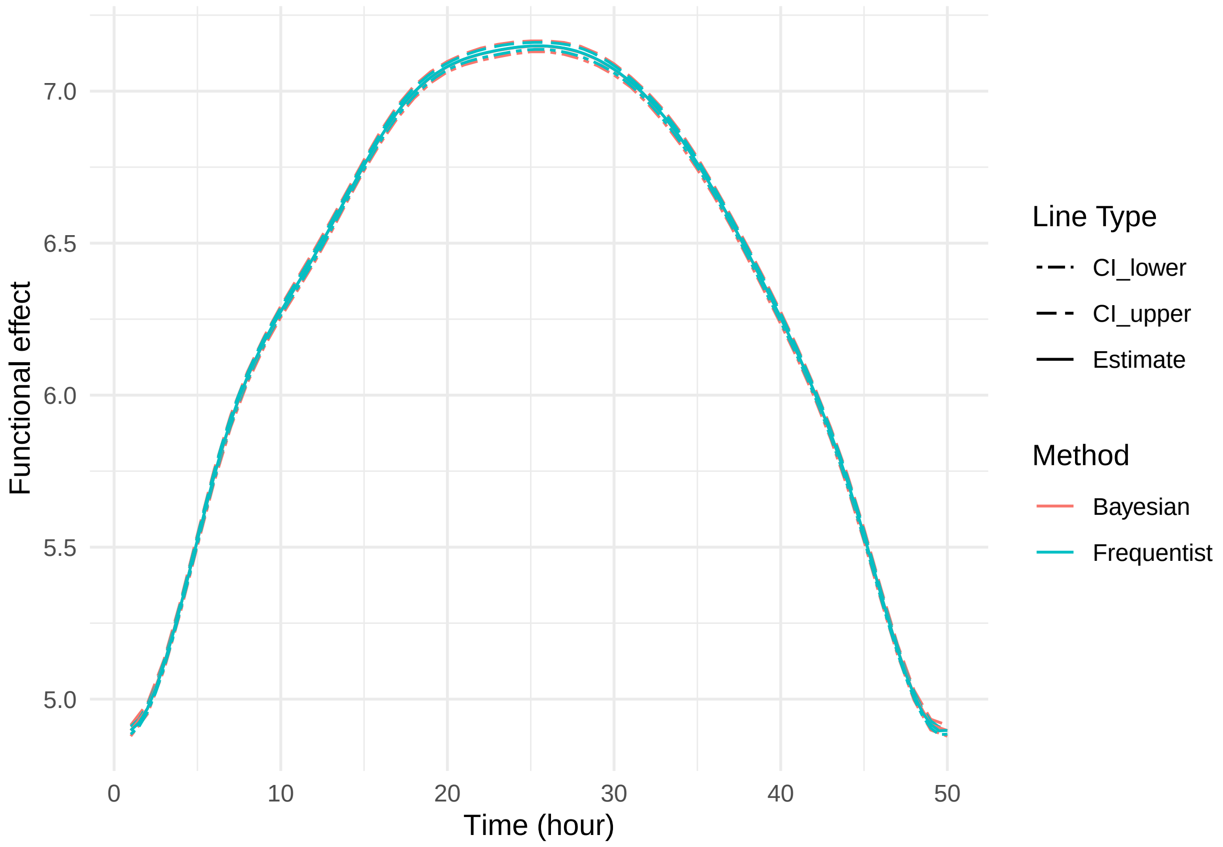

R code for fitting the equivalent model using refundBayes.

```
refundBayes_FunCox = refundBayes::bfrs(y ~ X,
      data = FoSR_exp_data,
```

```
      func_parameter = list(type = "cc", k=10),
      family = "functional",
      runStan = TRUE, # Whether automatically run Stan program.
      n.iter = 2000, # Total number of posterior sampling.
      n.warmup = 1000, # Burn-in value.
      n.knots = 3 # Number of parallel computed chains for posterior sampling.
      )
```

```
## Warning: The largest R-hat is NA, indicating chains have not mixed.
## Running the chains for more iterations may help. See
## https://mc-stan.org/misc/warnings.html#r-hat

## Warning: Bulk Effective Samples Size (ESS) is too low, indicating posterior means and medians may be
## Running the chains for more iterations may help. See
## https://mc-stan.org/misc/warnings.html#bulk-ess

## Warning: Tail Effective Samples Size (ESS) is too low, indicating posterior variances and tail quant
## Running the chains for more iterations may help. See
## https://mc-stan.org/misc/warnings.html#tail-ess
```

```
cat(refundBayes_FunCox$Stancode)
```

```
## data{
##    //Total number of observations
##    int<lower=1> N_num;
##    //Number of observed time points
##    int<lower=1> T;
##    //Functional outcome for FoSR
##    matrix[N_num, T] Y;
##    //Number of FPCA eigenfunctions
##    int ncol_phi;
##    //Matrix of FPCA eigenfunctions
##    matrix[ncol_phi, T] phi;
##    //Number of spline basis
##    int ncol_psi_mat;
##    //Matrix of spline basis
##    matrix[ncol_psi_mat, T] psi_mat;
##    //Penalty matrix
##    matrix[ncol_phi, ncol_phi] S_beta;
##    //Number of scalar predictors
##    int<lower=1> K_num;
##    //Matrix of scalar predictors
##    matrix[N_num,K_num] Z_mat;
## }
## transformed data {
## }
## parameters{
##    //Linear predictor intercept
##    real eta_0;
##    //FPCA scores
##    matrix[N_num,ncol_psi_mat] zxi;
##    real<lower=0> sigma_epis;
```

```
##    //Smoothing parameters
##    vector<lower=0>[K_num] sigma_b;
##    vector<lower=0>[ncol_psi_mat] sigma_k;
##    //Functional effect spline coefficients
##    matrix[K_num,ncol_phi] b;
## }
## transformed parameters {
##    real lprior = 0;
## }
## model{
##    //Linear predictor
##    matrix[N_num, T] mu;
##    matrix[K_num, T] betaT;
##    betaT = b *phi;
##    mu = Z_mat * betaT + zxi * psi_mat;
##    target += -N_num*T*log(sigma_epis)/2- sum((mu-Y)^2)/(2*sigma_epis^2);
##    for(nj in 1:ncol_psi_mat){
##      target += -N_num*log(sigma_k[nj])/2- sum((zxi[,nj])^2)/(2*sigma_k[nj]^2);
##      target += inv_gamma_lpdf(sigma_k[nj]^2|0.005,0.005);
##    }
##    for(nk in 1:K_num){
##      target += (- b[nk,]*S_beta* b[nk,]') / (2*sigma_b[nk]^2);
##      target += inv_gamma_lpdf(sigma_b[nk]^2|0.005,0.005);;
##    };
##    target +=  inv_gamma_lpdf(sigma_epis^2|0.005,0.005);
## }
```

```
plot.bfrs(refundBayes_FunCox)
```

```
## Warning in geom_line(aes(type = type, color = type)): Ignoring unknown
## aesthetics: type
```

```
## [[1]]
```

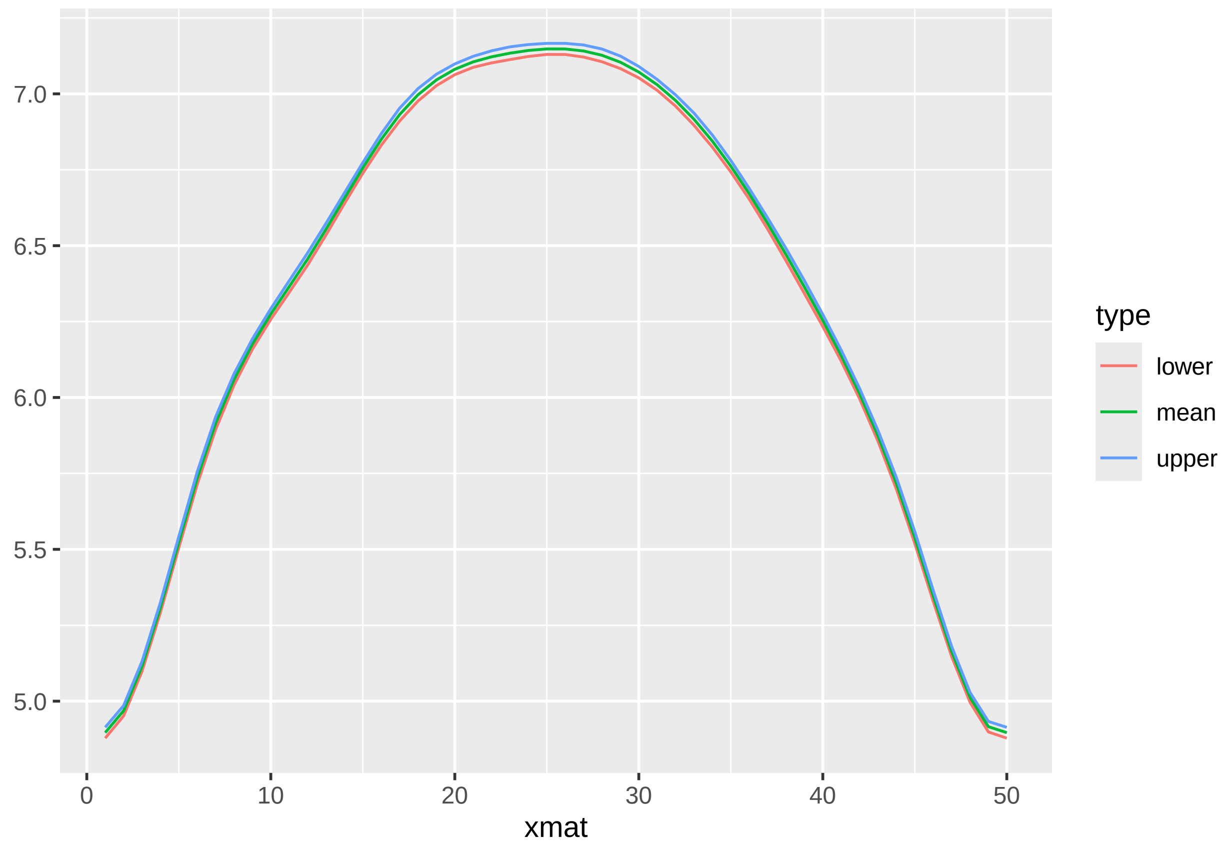

7.0
6.5
6.0
5.5
5.0
0
10
20
30
40
50
xmat
type
lower
mean
upper
