# Supplementary material for: Tutorial on Bayesian Functional Regression Using Stan
Source: Stat Med. 2025 Sep 14;44(20-22):e70265. doi: 10.1002/sim.70265 (PMC12433833; doi:10.1002/sim.70265)
Supplement: Supplementary file 1 — Data S1. Supporting Information. [file SIM-44-0-s001.zip › Supplementary materials/Bayesian_FDA_with_Stan_supp.pdf]

Received: Added at production | Revised: Added at production | Accepted: Added at production

DOI: xxx/xxxx

ARTICLE TYPE

Supplementary materials for "Tutorial on Bayesian Functional Regression Using Stan"

Ziren Jiang[1] | Ciprian Crainiceanu[2] | Erjia Cui[1]

[1]Division of Biostatistics and Health Data Science, University of Minnesota, MN, United States

[2]Department of Biostatistics, Johns Hopkins University, MD, United States

Correspondence
Corresponding author Erjia Cui
Email: ecui@umn.edu

Abstract

This manuscript provides step-by-step instructions for implementing Bayesian functional regression models using Stan. Extensive simulations indicate that the inferential performance of the methods is comparable to that of state-of-the-art frequentist approaches. However, Bayesian approaches allow for more flexible modeling and provide an alternative when frequentist methods are not available or may require additional development. Methods and software are illustrated using the accelerometry data from the National Health and Nutrition Examination Survey (NHANES).

1 | TIME CONSUMPTION IN SIMULATION

The following table summarizes the mean computation time (in minutes) for each method under our first simulation experiment, based on generating a total of 15,000 posterior samples. All simulations were performed using a single core of a 2.45 GHz CPU. The time includes the Stan model compiling and sampling.

TABLE 1 Time used (in minutes) for the Stan model with 15,000 posterior samples.

	**n=100**	**n=300**	**n=500**	**n=700**
$\tau = 1$	1.617	1.733	1.798	1.768
$\tau = 2$	1.542	1.727	1.967	1.982
$\tau = 3$	1.683	1.786	1.730	2.063
$\tau = 5$	1.569	1.754	1.730	2.277

2 | DATA-GENERATING MECHANISM FOR GENERATING SURVIVAL OUTCOME

We consider the total of $n = 100, 200, 300$, and 500 subjects and one functional covariate observed at $T = 50$ equally-spaced points in $[0, 1]$. The functional covariates $\{W_i(t_j), t_j \in [0, 1]\}_{t=1}^{T}$ are generated using the first 7 principal components estimated from our NHANES case study. The functional coefficient is $\beta(t) = \left(0.084 - (t - 0.5)^2\right) \times \tau$, $t \in [0, 1]$, with $\tau = 1, 2, 3$, and 5 controlling the strength of the signal.

The Survival outcomes were simulated by inverting the survival function derived from the cumulative baseline hazard $\hat{H}_0(t)$ estimated from our case study, smoothed using a monotonic P-spline (`scam` with `bs = "mpi"`). For each subject, survival time T_i was drawn by solving $S_i(t) = \exp\left\{-\Lambda_0(t)\exp(\eta_i)\right\} = U_i$, where $U_i \sim \text{Unif}(0, 1)$ and η_i is the subject-specific linear predictor. Independent censoring times were sampled from the empirical distribution of simulated event times to mimic real-world censoring. The observed event time was then defined as $\min(T_i, C_i)$ with the corresponding censoring indicator.

 © 2023 Copyright Holder Name

3 | ADDITIONAL RESULTS FOR CASE STUDY

In the supplementary material, we present additional results for our NHANES example in Section 6.

3.1 | Bayesian model diagnostic

To examine the convergence of the posterior sampling, we call the `traceplot` function from `R Stan` package for the trace plot, which is displayed in Figure 1 of the supplementary materials. The trace plot indicates a good convergence of and agreement among the posterior sample chains.

```
// Trace plot for Bayesian posterior samples
traceplot(fit_bfrs$stanfit,pars=c("Intercept","bs","zbr_1"))
```

FIGURE 1 Estimated functional effect for the scalar-on-function regression for Bayesian (left) and Frequentist (right) methods. Darker gray shaded area bordered by dashed lines: pointwise 95% confidence/credible interval. Lighter gray shaded area bordered by dotted lines: CMA 95% confidence/credible interval.

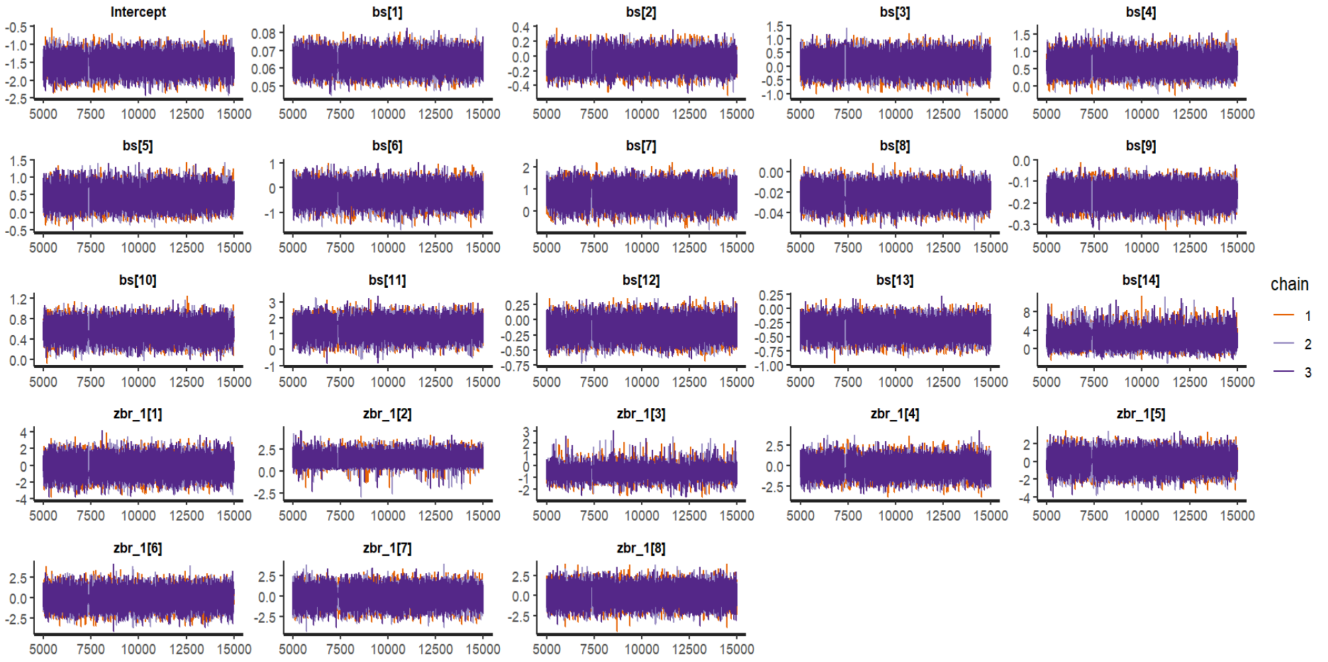

3.2 | Bayesian jointly modelling of FPCA

In addition to the functional Cox regression model, we also addressed measurement error using functional PCA, as described in Section 4 of the main manuscript. We compared our Bayesian joint modeling approach to a frequentist two-step procedure. As noted in the manuscript, the two-step approach does not account for the uncertainty in the FPCA-fitted functional predictor, which may lead to an underestimation of the variability in the estimated functional coefficient.

The computational burden of the Bayesian joint model is substantially higher than that of the Bayesian regression model. To reduce computation time, we downsampled the observation grid from 1440 time points per day to 72. Despite this reduction,

running the Stan program to obtain 2000 posterior samples with 3 parallel chains still required approximately 20 hours on a personal computer with a 4 GHz processor.

The estimated functional coefficients are displayed in Figure 2. The two approaches yield similar point estimates, but the Bayesian joint model produces noticeably wider credible intervals, reflecting its incorporation of uncertainty in the functional predictor. The estimated scalar coefficients are presented in Table 2, where the two approaches have very similar results.

FIGURE 2 Estimated functional effect for the scalar-on-function regression for Bayesian and Frequentist methods. We also provide the pointwise 95% confidence/credible interval for each method.

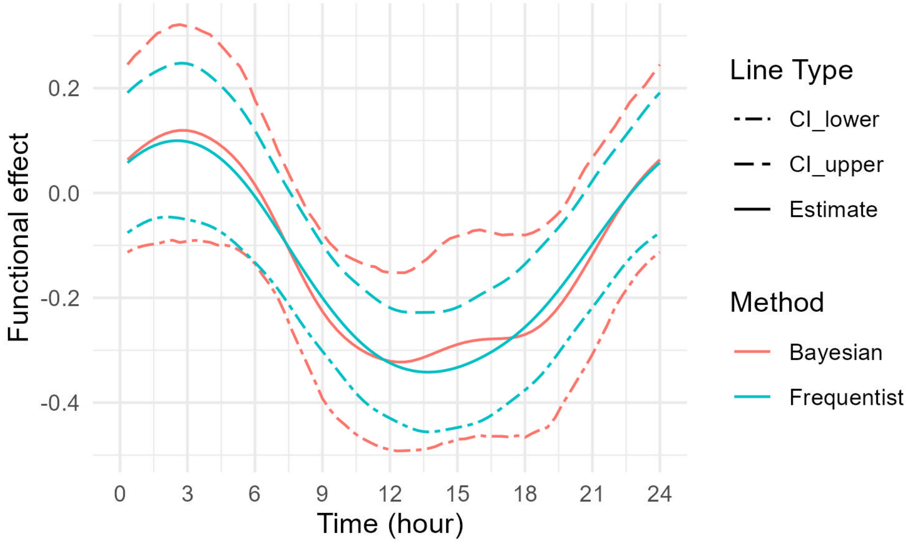

4 | SOFTWARE DEVELOPMENT

The `refundBayes` R package is available at https://github.com/ZirenJiang/refundBayes.

TABLE 2 Estimated coefficients for scalar predictors in the NHANES example with corresponding 95% confidence/credible intervals for the frequentist and Bayesian implementations with FPCA.

**Scalar Covariate**	**Bayesian Estimate**	**Frequentist Estimate**
Age	0.066 (0.055 , 0.073)	0.066 (0.059 , 0.073)
Gender (Female)	-0.069 (-0.225 , 0.090)	-0.074 (-0.225 , 0.077)
BMI	-0.023 (-0.035 , -0.012)	-0.023 (-0.039 , -0.007)
PIR	-0.162 (-0.218 , -0.105)	-0.163 (-0.240 , -0.087)
Race		
Other Hispanic	0.144 (-0.573 , 0.280)	-0.139 (-0.553 , 0.275)
Non-Hispanic White	0.323 (0.014 , 0.651)	0.313 (0.000 , 0.627)
Non-Hispanic Black	0.219 (-0.107 , 0.563)	0.212 (-0.116 , 0.541)
Non-Hispanic Asian	-0.445 (-0.907 , 0.017)	-0.438 (-0.900 , 0.023)
Other Race	0.482 (-0.096 , 1.028)	0.500 (-0.048 , 1.048)
CHD		
Yes	0.440 (0.228 , 0.640)	0.437 (0.230 , 0.644)
Don't know	0.614 (-0.005 , 1.169)	0.650 (0.057 , 1.243)
Education		
High school equivalent	-0.061 (-0.259 , 0.133)	-0.063 (-0.259 , 0.134)
More than high school	-0.390 (-0.199 , -0.013)	-0.203 (-0.393 , -0.013)
Don't know	0.525 (-0.889 , 1.650)	0.667 (-0.510 , 1.844)
